# Hybrid clone cells derived from human breast epithelial cells and human breast cancer cells exhibit properties of cancer stem/initiating cells

**DOI:** 10.1186/s12885-017-3509-9

**Published:** 2017-08-02

**Authors:** Daria Gauck, Silvia Keil, Bernd Niggemann, Kurt S. Zänker, Thomas Dittmar

**Affiliations:** 1 Institute of Immunology & Experimental Oncology, Center for Biomedical Education and Research (ZBAF), Witten/Herdecke University, Stockumer Str. 10, 58448 Witten, Germany; 2 Technical University Dortmund, Dortmund, Germany

**Keywords:** Cell fusion, Mammosphere formation capacity, Colony formation capacity, ALDH1-positive cancer cells, Breast cancer, Cancer stem/initiating cells, Cell migration

## Abstract

**Background:**

The biological phenomenon of cell fusion has been associated with cancer progression since it was determined that normal cell × tumor cell fusion-derived hybrid cells could exhibit novel properties, such as enhanced metastatogenic capacity or increased drug resistance, and even as a mechanism that could give rise to cancer stem/initiating cells (CS/ICs). CS/ICs have been proposed as cancer cells that exhibit stem cell properties, including the ability to (re)initiate tumor growth.

**Methods:**

Five M13HS hybrid clone cells, which originated from spontaneous cell fusion events between M13SV1-EGFP-Neo human breast epithelial cells and HS578T-Hyg human breast cancer cells, and their parental cells were analyzed for expression of stemness and EMT-related marker proteins by Western blot analysis and confocal laser scanning microscopy. The frequency of ALDH1-positive cells was determined by flow cytometry using AldeRed fluorescent dye. Concurrently, the cells’ colony forming capabilities as well as the cells’ abilities to form mammospheres were investigated. The migratory activity of the cells was analyzed using a 3D collagen matrix migration assay.

**Results:**

M13HS hybrid clone cells co-expressed SOX9, SLUG, CK8 and CK14, which were differently expressed in parental cells. A variation in the ALDH1-positive putative stem cell population was observed among the five hybrids ranging from 1.44% (M13HS-7) to 13.68% (M13HS-2). In comparison to the parental cells, all five hybrid clone cells possessed increased but also unique colony formation and mammosphere formation capabilities. M13HS-4 hybrid clone cells exhibited the highest colony formation capacity and second highest mammosphere formation capacity of all hybrids, whereby the mean diameter of the mammospheres was comparable to the parental cells. In contrast, the largest mammospheres originated from the M13HS-2 hybrid clone cells, whereas these cells’ mammosphere formation capacity was comparable to the parental breast cancer cells. All M13HS hybrid clones exhibited a mesenchymal phenotype and, with the exception of one hybrid clone, responded to EGF with an increased migratory activity.

**Conclusion:**

Fusion of human breast epithelial cells and human breast cancer cells can give rise to hybrid clone cells that possess certain CS/IC properties, suggesting that cell fusion might be a mechanism underlying how tumor cells exhibiting a CS/IC phenotype could originate.

**Electronic supplementary material:**

The online version of this article (doi:10.1186/s12885-017-3509-9) contains supplementary material, which is available to authorized users.

## Background

The biological phenomenon of cell fusion plays a crucial role in several physiological functions, such as fertilization, tissue regeneration and wound healing, as well as pathophysiological conditions, including cancer (for review see: [1, 2]). In vitro and in vivo data have demonstrated that the fusion of tumor cells with other tumor cells or tumor cells with normal cells, such as macrophages, stromal cells, fibroblasts and stem cells, could give rise to unique hybrid cells that exhibit novel properties such as enhanced metastatic capacity or increased drug resistance [3–13]. For instance, Rachkovsky and colleagues demonstrated that most human macrophage × Cloudman S91 melanoma cell hybrids were more aggressive than the parental melanoma cells and produced metastases sooner and in more mice [9]. Analysis of a cell line derived from a human breast adenocarcinoma xenograft revealed that approximately 30% of the cells had mixed mouse and human chromosomes, among which 8% carried mouse/human translocations, indicating that the hybrid cells originated by spontaneous fusion between the malignant human epithelium and normal host mouse stroma [6]. Such transformed stroma-derived cells were tumorigenic with histopathologic features of malignancy, suggesting the impact of cell fusion in tumor progression [6]. Using a parabiosis animal model, Powell and colleagues were able to demonstrate that massive cell fusion events occurred in the tumorigenic intestine of an APC^Min−/−^/ROSA26 mouse that was surgically joined to a GFP mouse [8]. Gene expression studies of such cell fusion hybrids showed that they retained transcriptome characteristics from both parental lineages, while also developing an additional novel transcriptome profile, unique from either parental lineage [8]. Moreover, a number of genes known to be modulated in metastasis were transcriptionally altered within the hybrid cells, supporting the hypothesis that cancer cells could acquire metastatic capabilities through cell fusion [8]. Using differentially labeled SKBR3 breast cancer cells, Yan and colleagues were able to demonstrate that chemotherapy promotes cell fusion in vivo [13]. Treatment of mice with Epirubicin was correlated with an increased frequency of hybrid cells (up to 12%) in the outer section of the tumor [13], indicating that chemotherapy might promote the origin of drug-resistant cancer hybrid cells.

In addition to an enhanced metastatic capacity and an increased drug resistance, cancer hybrid cells may also exhibit cancer stem/initiating cell (CS/IC) properties [14–16]. Thereby, the fusion of a stem cell (which may already possess chromosomal aberrations) and a somatic cell (which may already possess chromosomal aberrations) or a cancer cell could give rise to a genomic instable hybrid cell putatively exhibiting CS/IC properties [14]. This is consistent with data demonstrating that CD34 liver cancer stem cells were formed by fusion of hepatobiliary stem/progenitor cells with hematopoietic precursor-derived myeloid intermediates [17]. Likewise, fusion of embryonic stem cells with hepatocellular carcinoma cells gave rise to hybrid cells that were similar to liver tumor-initiating cells [18]. Hybrid cells derived from human umbilical cord mesenchymal stem cells (hucMSCs) and gastric cancer cells exhibited an epithelial-mesenchymal-transition (EMT) phenotype [19]. Moreover, hybrid cells revealed an increased expression of both types of markers: the stemness factors OCT4, NANOG, SOX2 and LIN28, as well as the cancer stem cell marker CD133, which was further correlated to an increased tumorigenic capacity in a xenograft model [19]. Similarly, hybrid cells derived from spontaneously fused non-small cell lung cancer cells and bone marrow-derived mesenchymal stem cells exhibited an increased expression of the stem cell marker CD133 and overexpression of stemness factors, including OCT4, NANOG, BMI-1, NOTCH1, ALDH1 and SOX2, which was associated with an increased pneumosphere-forming capacity and tumor-forming ability [20]. Rappa et al. demonstrated that spontaneously formed hybrid cells derived from human MA11 and MDA-MB-231 breast cancer cells and human bone marrow-derived multipotent stromal cells were both tumorigenic and metastatogenic in immunodeficient mice [11]. Given that only CS/ICs are capable of inducing primary tumor formation, which also applies to metastases [16] and cancer relapses [15], the increased tumorigenic and metastatic capabilities of MDA hybrids may indicate inherent CS/IC properties [11]. However, even in the absence of chromosomal aberrations, the fusion of two non-tumorigenic epithelial cells could give rise to highly tumorigenic hybrid cells [21], most likely as a consequence of the cell fusion-induced genomic instability.

In previous studies, we have already demonstrated that human M13SV1-EGFP-Neo breast epithelial cells that exhibit stem-like characteristics and human HS578T-Hyg breast cancer cells spontaneously fuse with each other, thereby giving rise to individual M13HS hybrid clones possessing unique properties, such as an enhanced drug resistance and an altered migratory behavior [15, 22, 23]. Because the fusion of a cancer cell with a stem cell (or a stem-like cell) may result in CS/IC-like hybrid cells, M13HS hybrid cell clones were analyzed for CS/IC-related characteristics, including the expression of stemness factors SOX9 and SLUG, the capacity of forming colonies and mammospheres, as well as aldehyde dehydrogenase 1 (ALDH1) expression. In brief, our data show that M13HS hybrid clones exhibit certain CS/IC properties, suggesting that CS/ICs could originate from cell fusion events.

## Methods

### Cell culture

M13SV1-EGFP-Neo cells were derived from M13SV1-EGFP-Neo human breast epithelial cells (a kind gift of James Trosko, Michigan State University, East Lansing, MI [24]) and were stably transfected with the pEGFP-NEO vector [22]. Cells were cultured in MSU-1 media (Biochrom GmbH, Berlin, Germany) supplemented with 10% fetal calf serum (FCS; Biochrom GmbH, Berlin, Germany), 100 U/mL penicillin/ 0.1 mg/mL streptomycin (Sigma-Aldrich, Taufkirchen, Germany), 10 μg/mL epidermal growth factor (EGF; human recombinant), 5 μg/mL Insulin (human recombinant), 0.5 μg/mL hydrocortisone, 4 μg/mL transferrin (human), 10 nM β-estrogen (all supplements were purchased from Sigma-Aldrich, Taufkirchen, Germany), and 400 μg/mL G418 (Biochrom GmbH, Berlin, Germany). HS578T-Hyg human breast cancer cells were derived from HS578T cells (HTB 126; LGC Standards GmbH, Wesel, Germany) by stable transfection with the pKS-Hyg vector. Cells were cultured in RPMI 1640 media (Sigma Aldrich, Taufkirchen, Germany) supplemented with 10% FCS (Biochrom GmbH, Berlin, Germany), 100 U/mL penicillin, 0.1 mg/mL streptomycin (Sigma-Aldrich, Taufkirchen, Germany), and 200 μg/mL hygromycin B (Pan Biotech, Aidenbach, Germany). M13HS-X hybrid clone cells (X = 1, 2, 4, 7, 8) [15, 22] were cultivated in RPMI 1640 media (Sigma Aldrich, Taufkirchen, Germany) supplemented with 10% FCS (Biochrom GmbH, Berlin, Germany), 100 U/mL penicillin, 0.1 mg/mL streptomycin (Sigma-Aldrich, Taufkirchen, Germany), 400 μg/mL G418 (Biochrom GmbH, Berlin, Germany) and 200 μg/mL hygromycin B (Pan Biotech, Aidenbach, Germany).

### Cultivation of mammospheres

Mammospheres were generated by seeding cells (3×10^5^ cells in 6 mL medium) in DMEM (high glucose; Sigma-Aldrich, Taufkirchen, Germany) supplemented with 6.6% B27 (Thermo Fisher Scientific, Bonn, Germany), 100 U/mL penicillin, 0.1 mg/mL streptomycin (Sigma-Aldrich, Taufkirchen, Germany), 20 ng/mL fibroblast growth factor (FGF; human recombinant; Sigma-Aldrich, Taufkirchen, Germany) and 20 ng/mL EGF (human recombinant; Sigma-Aldrich, Taufkirchen, Germany) in ultralow adherent cell culture flasks (Sarstedt AG&Co, Nürmbrecht, Germany) in a humidified atmosphere at 37 °C and 5% CO_2_. For cultivation of HS578T-Hyg mammospheres, 200 μg/mL hygromycin B (Pan Biotech, Aidenbach, Germany), and for M13HS mammospheres 400 μg/mL G418 (Biochrom GmbH, Berlin, Germany) and 200 μg/mL hygromycin B (Pan Biotech, Aidenbach, Germany) was added. Mammospheres were cultured for up to 10 days.

### Western blot analysis

Cells were harvested, washed once with phosphate-buffered saline (PBS) and adjusted to a cell number of 2×10^5^ cells/20 μL. Subsequently, 10 μL of 3× Laemmli sample buffer was added and samples were lysed for 10 min at 95 °C. Depending on the protein of interest, samples were separated by 10 or 12% sodium dodecyl sulfate-polyacrylamide gel electrophoresis (SDS-PAGE) and transferred to an Immobilon polyvinyl difluoride (PVDF) nitrocellulose membrane (Merck Millipore, Darmstadt, Germany) under semi-dry conditions. Membranes were blocked with 10% (*w*/*v*) non-fat milk powder or 5% bovine serum albumin (BSA) in Tris-buffered saline with 1% Tween 20 (TBS-T). Bands were visualized using the Pierce ECL Western blot substrate (Thermo Fisher Scientific, Bonn, Germany) in accordance to the manufacturer’s instructions and the Aequoria Macroscopic Imaging System (Hamamatsu Photonics Germany, Herrsching am Ammersee, Germany). Antibodies used for Western blot analysis are listed in Table 1.

**Table 1:** Summary of antibodies used in this study

Antibody	Manufacturer
SOX9; rabbit polyclonal	Santa Cruz^b^
*SOX9; rabbit monoclonal #D8G8H* ^a^	Cell Signaling^c^
SLUG; rabbit monoclonal #C19G7	Cell Signaling^c^
*SLUG; mouse monoclonal #C15D3* ^a^	Becton Dickenson^d^
SNAIL; rabbit monoclonal #C15D3	Cell Signaling^c^
E-CADHERIN; rabbit monoclonal #24E10	Cell Signaling^c^
N-CADHERIN; mouse monoclonal #32	Becton Dickenson^d^
TWIST; mouse monoclonal #Twist2C1a	Abcam^e^
VIMENTIN; rabbit monoclonal #R28	Cell Signaling^c^
CK8; mouse monoclonal #H-11	Santa Cruz^b^
CK14; goat polyclonal	Santa Cruz^b^
ZEB1; rabbit monoclonal #D80D3	Cell Signaling^c^
ZEB2; rabbit polyclonal	Abcam^e^
elf4E; rabbit monoclonal	Cell Signaling^c^
β-actin; rabbit monoclonal #13E5	Cell Signaling^c^
anti-mouse-IgG-HRP-linked	Cell Signaling^c^
anti-rabbit-IgG-HRP-linked	Cell Signaling^c^
*goat-anti-mouse-Cy3* ^a^	Jackson ImmunoResearch^f^
*goat-anti-rabbit-Cy5* ^a^	Jackson ImmunoResearch^f^

^a^These antibodies were used for confocal laser scanning microscopy

^b^Santa Cruz Biotechnology, Heidelberg, Germany

^c^New England Biolabs GmbH, Frankfurt am Main, Germany

^d^Becton Dickenson Laboratories, Heidelberg, Germany

^e^Abcam, Cambridge, United Kingdom

^f^Jackson ImmunoResearch Europe Ltd., Dianova, Hamburg, Germany

### Colony-forming assay

Cells (2×10^2^ per well) were seeded in 6-well plates and were cultivated in complete media for 10–14 days. Subsequently, media was removed, cells were washed twice in PBS and were fixed and stained with 6% (*v*/v) glutaraldehyde and 0.5% crystal violet (both reagents were purchased from Sigma-Aldrich, Taufkirchen, Germany) for 60 min at room temperature. Plates were thoroughly washed with water and air-dried at room temperature.

### Mammosphere -formation assay

Prior to cultivation, 96-well plates were first coated with 50 μL poly-(2-hydroxyethyl-methacrylate) (poly-HEMA; 1.2% (*w*/*v*) in ethanol; Sigma-Aldrich, Taufkirchen, Germany). Plates were maintained for up to 3 days in the incubator at 37 °C, allowing the ethanol to evaporate completely. Cells were harvested and seeded at a density of 5×10^2^ cells per well of a 96-well plate in mammosphere formation medium (80% medium I [40% (*v*/v) Methocult H4100 (Stem Cells Technologies, Cologne, Germany) and 60% (*v*/v) DMEM (Sigma-Aldrich, Taufkirchen, Germany)] and 20% medium II [MammoCult Human Medium (Stem Cells Technologies, Cologne, Germany]) supplemented with 20 ng/mL FGF (human recombinant; Sigma-Aldrich, Taufkirchen, Germany), 20 ng/mL EGF (human recombinant; Sigma-Aldrich, Taufkirchen, Germany) and 0.39 μg/mL hydrocortisone (Sigma-Aldrich, Taufkirchen, Germany). After 10 days in culture, the size and the number of mammospheres grown were determined by video microscopy. Mammospheres with a diameter < 60 μm were excluded from analysis.

### AldeRed assay

The AldeRed aldehydedehydrogenase 1 (ALDH1) assay (Merck Millipore, Darmstadt, Germany) was performed in accordance with the manufacturer’s instructions. In brief, 2×10^5^ cells were resuspended in AldeRed assay buffer containing the AldeRed 588-A substrate. The cell suspension was divided into two fractions, whereby one half served as a control, and transferred to a new tube containing the specific ALDH1 inhibitor diethylamino benzaldehyde (DEAB). Cells were incubated for 30 min at 37 °C in the dark. Subsequently, cells were centrifuged (300×*g*, 5 min), the supernatant was discarded, and the cell pellet was resuspended in 500 μL of AldeRed assay buffer. Samples were stored on ice prior to flow cytometry (FACSCalibur; Becton Dickenson, Heidelberg, Germany). FACS data were analyzed using WinMDI 2.9.

### Confocal laser scanning microscopy

The expression and distribution of SOX9 and SLUG within single cells and mammospheres were visualized by confocal laser scanning microscopy (Leica TCS SP5; Leica Microsystems, Wetzlar, Germany). For single-cell analysis, cells (1×10^4^) were seeded in chamber slides (Nunc Lab-Tek; Thermo Fisher Scientific, Germany) in the appropriate media for 24 to 48 h in a humidified atmosphere at 37 °C and 5% CO_2_. Cells were fixed with paraformaldehyde (PFA; 4% [*w*/*v*] in PBS; 20 min, room temperature [RT]), washed twice with PBS and were permeabilized with 1% Triton X-100 ([*v*/v] in PBS; 5 min, RT). Subsequently, cells were washed again twice with PBS and were then incubated with BSA-solution (1.5% BSA [*w*/*v*] in PBS) to block unspecific binding sites. Samples were stained with specific antibodies against SLUG (mouse monoclonal; Table 1) and SOX9 (rabbit monoclonal; Table 1) diluted in BSA-solution for 60 min at RT. Thereafter, samples were washed three times with BSA-solution and were then incubated with secondary goat-anti-mouse-Cy3 and goat-anti-mouse-Cy5 antibodies (Table 1) diluted in BSA-solution for 60 min at RT in the dark. Cells were washed again three times with BSA-solution and were then incubated with SYTOX-Green (Thermo Fisher Scientific, Bonn, Germany) for 15 min at RT in the dark for nuclear staining. After thorough washing (three times with BSA-solution), samples were mounted with Fluoromount (Sigma-Aldrich, Taufkirchen, Germany). For analysis, the DNA stain was colored in blue, SOX9 in green and SLUG in red. Images were processed using ImageJ (imagej.nih.gov/ij/).

Mammospheres were cultured as described above and were then transferred to the upper compartment of a Boyden chamber (pore size 4 μm; Sarstedt AG&Co, Nürmbrecht, Germany) within a 24-well plate (Sarstedt AG&Co, Nürmbrecht, Germany) and washed three times with PBS. Subsequently, mammospheres were fixed and permeabilized with 4% PFA (in PBS) and 1% Triton ([*v*/v] in PBS) for at least 3 h at 4 °C. To ensure that cells within deeper areas of the mammospheres were also permeabilized, a methanol-based permeabilization method was applied by incubating the mammospheres in increasing concentrations of ice-cold methanol diluted in PBS (25, 50, 75, 95 and 100%). Each incubation was carried out at 4 °C and lasted 30 min. Subsequently, samples were rehydrated by incubating them in decreasing concentrations of ice-cold methanol diluted in PBS (95, 75, 50, and 25%) at 4 °C (each incubation step lasted 30 min). Mammospheres were washed three times with PBS. To avoid unspecific staining, fixed and permeabilized mammospheres were incubated with 3% BSA (*w*/*v*) and 0.1% Triton (*v*/v) in PBS at 4 °C overnight. Mammospheres were washed twice with PBS and were then stained for SOX9 and SLUG using the antibodies described above (see also Table 1) for 48 h under gentle agitation at 4 °C. Subsequently, mammospheres were washed four times with PBS and were then incubated with Cy3- and Cy5-conjugated secondary antibodies (see above and Table 1) for 24 h under gentle agitation at 4 °C. Mammospheres were washed again three times with PBS and were then incubated with SYTOX-Green (Thermo Fisher Scientific) for 2 h at RT in the dark. Finally, samples were washed again three times with PBS and were then mounted with Fluoromount (Sigma-Aldrich, Taufkirchen, Germany). For analysis, the DNA stain was colored in blue, SOX9 in green and SLUG in red. Images were processed using ImageJ (imagej.nih.gov/ij/).

### Karyotype analysis

Parental cells and M13HS hybrid clone cells (5×10^6^) were cultured for 4 h with 0.2 μg/ml Colcemid solution (KaryoMax Colcemid Solution; Thermo Fisher Scientific, Bonn, Germany) in a humidified atmosphere at 37 °C and 5% CO_2_. Subsequently, cells were harvested, washed once with PBS and were resuspended in 75 mM KCl for 30 min. Cells were fixed in methanol and acetic acid (3:1) and were carefully washed twice with methanol/ acetic acid (3:1). Pipette three drops of the cell suspension onto a clean and wet slide and dry at room temperature. Finally, chromosomes were stained with SYTOX Green (Thermo Fisher Scientific, Bonn, Germany) and visualized by confocal laser scanning microscopy (Leica TCS SP5; Leica Microsystems, Wetzlar, Germany). Images were processed using ImageJ (imagej.nih.gov/ij/).

### Cell morphology

Parental cells and M13HS hybrid clone cells (2×10^4^) were seeded onto cover slips in the appropriate media for 24 h in a humidified atmosphere at 37 °C and 5% CO_2_. Cells were fixed with paraformaldehyde (PFA; 4% (*w*/*v*) in PBS; 20 min, room temperature), washed twice with PBS and were permeabilized with 1% Triton X-100 ((*v*/v) in PBS; 5 min, RT). Subsequently, cells were washed again twice with PBS and were stained with Phalloidin-Alexa568 (1 h, room temperature) and SYTOX Green (15 min, room temperature; both dyes from Thermo Fisher Scientific, Bonn, Germany). Samples were washed again two time with PBS, mounted with Fluoromount (Sigma-Aldrich, Taufkirchen, Germany) and were finally analyzed by confocal laser scanning microscopy (Leica TCS SP5; Leica Microsystems, Wetzlar, Germany). Images were processed using ImageJ (imagej.nih.gov/ij/).

### Cell migration studies

The analysis of the migratory activity within a 3D collagen matrix was performed as described [22, 25]. In brief, 4×10^6^ cells were resuspended in 50 μL medium, which was thoroughly mixed with 100 μL collagen solution composed of liquid collagen (PureCol; Nutacon BV, Leimuiden, The Netherlands), 10× minimal essential medium (Sigma-Aldrich, Taufkirchen, Germany) and 7.5% sodium bicarbonate solution (Sigma-Aldrich, Taufkirchen, Germany). EGF (final concentration 100 ng/mL) was added to the cell suspension. The collagen-cell suspension was filled in self-constructed migration chambers and the collagen was allowed to polymerize at 37 °C, 5% CO_2_ in the incubator. Subsequently, the migration chambers were filled with media, sealed with wax at the fourth site and were placed on a hot plate (adjusted to 37 °C) under a microscope. Cell migration was recorded for at least 15 h by time-lapse video microscopy. For analysis, 30 cells per condition were chosen randomly and the paths of the cells were tracked manually. The locomotor activity of the analyzed cell population is displayed as a box plot diagram and indicates the mean locomotor activity of 50% of the tracked cells within the observation period. The parameter “time active” is shown as a bar chart and summarizes the total time a single cell was moving within the observation period. Non-moving cells possess a time active of 0%. A time active of 20%, for example, indicates that the total time a particular cell migrated was between 1 and 180 min. A detailed explanation of the cell migration assay used, data acquisition and analysis, including video tutorials, is given in Rommerswinkel et al. [25].

### Statistical analysis

Statistical analysis was performed using an unpaired, two-tailed Student’s t-test. The two-tailed Mann-Whitney U test was used for statistical analysis of cell migration data.

## Results

### Hybrid cells derived from human breast epithelial cells and human breast cancer cells express mammary stemness factors

Cell fusion has been considered as a possible mechanism for how CS/ICs could evolve [14]. Since M13SV1-EGFP-Neo human breast epithelial cells exhibit stem-like properties [24], five distinct M13HS hybrid cell clones (M13HS-1, −2, −4, −7, and −8), which were derived from spontaneous cell fusion events between M13SV1-EGFP-Neo cells and human HS578T-Hyg breast cancer cells [15], were analyzed for the expression of SOX9 and SLUG, which cooperatively determine the mammary (cancer) stem cell state [26], and for the luminal and basal markers cytokeratin 8 (CK8) and CK14 [27]. M13HS hybrid cell clones cells are mono-nuclear, indicating that heterokaryon-to-synkaryon transition (HST) [14] has occurred and that the clones possess an increased mean chromosomal number, although this number varied among individual hybrid clones (Additional files 1 and 2) [15].

**Fig. 1:**
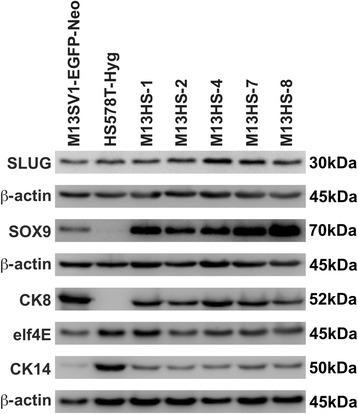
M13HS hybrid clone cells and parental cells reveal a differential expression of stemness-related marker proteins. M13HS hybrid clone cells reveal a joint expression pattern of SOX9, SLUG, CK8, and CK14, which are differentially expressed in the M13SV1-EGFP-Neo human breast epithelial cells and HS578T-Hyg human breast cancer cells. Shown are representative Western blot data of three independent experiments

M13SV1-EGFP-Neo human breast epithelial cells expressed high levels of both SLUG and CK8, whereas SOX9 was moderately expressed and only a faint expression of CK14 was detected (Fig. 1). In contrast, HS578T-Hyg human breast cancer cells were positive for SLUG and CK14 and negative for SOX9 and CK8 expression (Fig. 1). An overlap of the parental expression pattern of SOX9, SLUG, CK8 and CK14 was determined in all M13HS hybrid cell clones (Fig. 1). Interestingly, SOX9 levels were markedly higher in all M13HS hybrid cell clones compared to the M13SV1-EGFP-Neo breast epithelial cells (Fig. 1). Because SOX9 and SLUG are the determinants of a mammary (cancer) stem cell state [26], these findings may suggest that M13HS hybrid cell clones putatively exhibit CS/IC properties.

### Confocal laser scanning microscopy data

**Fig. 2:**
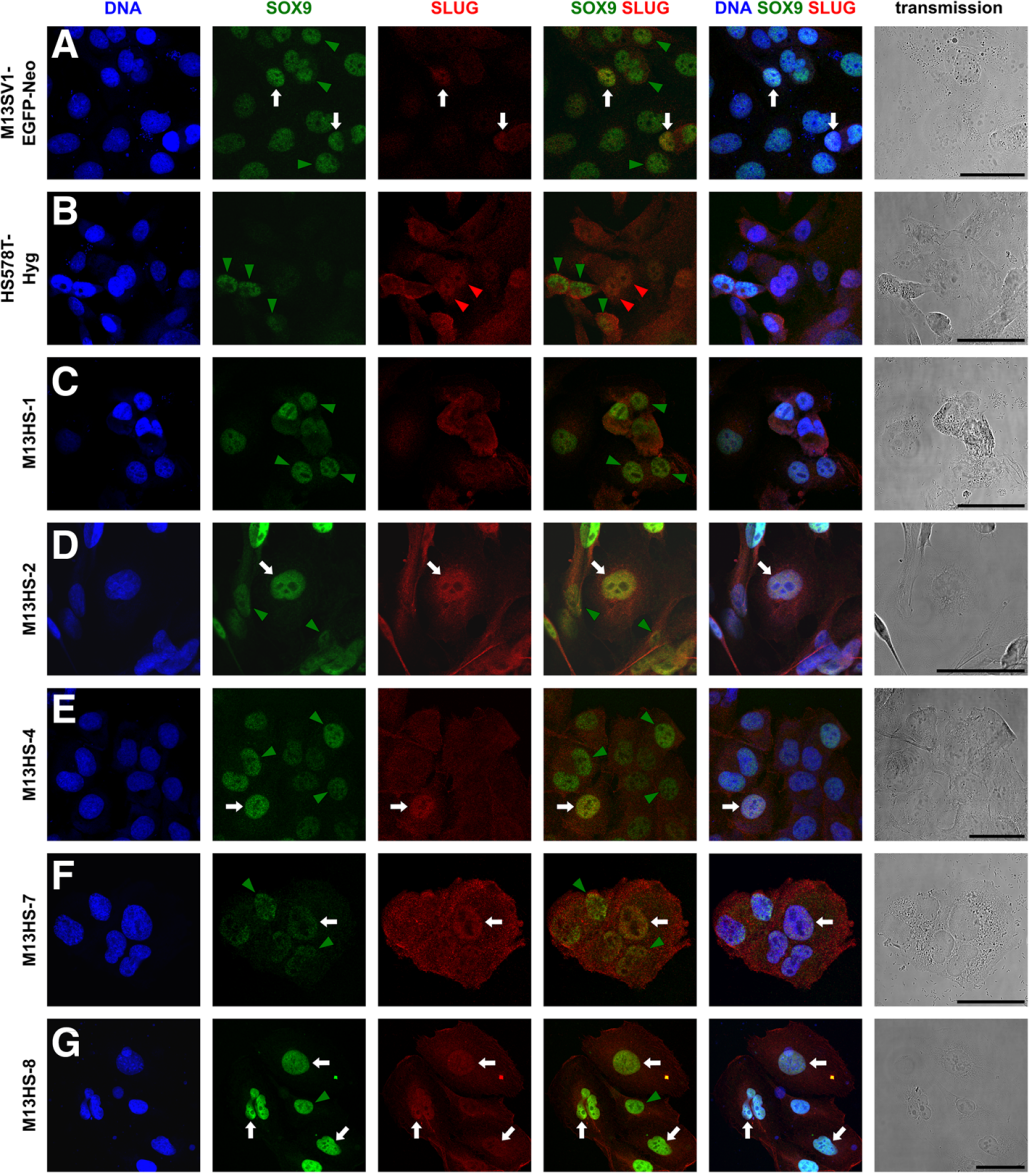
Confocal laser scanning microscopy of intracellular SOX9 and SLUG localization. **a** M13SV1-EGFP-Neo human breast epithelial cells, **b** HS578T-Hyg human breast cancer cells, **c** M13HS-1 hybrid cells, **d** M13HS-2 hybrid cells, **e** M13HS-4 hybrid cells, **f** M13HS-7 hybrid cells, **g** M13HS-8 hybrid cells. *White arrows* indicate cells with a nuclear co-localization of SOX9 and SLUG. Cells with SOX9 in the nucleus and SLUG in the cytoplasm are marked with a *green arrowhead*, whereas *red arrowheads* indicate cells with a nuclear localization of SLUG. Shown are data representative of three experiments. Bar = 50 μm

Western blot data of SOX9 and SLUG were validated by immunohistochemistry to visualize the intracellular distribution of both proteins (Fig. 2). In accordance with the Western blot analysis, SOX9 and SLUG expression was detected in M13SV1-EGFP-Neo breast epithelial cells (Fig. 2a). A nuclear co-localization of SLUG and SOX9 was observed in a few M13SV1-EGFP-Neo cells (Fig. 2a, white arrows), whereas in the majority of cells, SLUG was present in the cytosol (Fig. 2a, green arrowheads). In contrast, all HS578T-Hyg breast cancer cells were positive for SLUG expression, which was mainly distributed within the cytosol of the cells (Fig. 2b). However, in some HS578T-Hyg breast cancer cells, SLUG was also identified in the nucleus (Fig. 2b, red arrowheads). In contrast to the M13SV1-EGFP-Neo cells and in accordance with the Western blot data, SOX9 was solely expressed in a few HS578T-Hyg cells. Here, SOX9 was present in the nucleus (Fig. 2b, green arrowheads). Nuclear co-localization of both proteins in the HS578T-Hyg breast cancer cells was not observed. Co-expression of both SOX9 and SLUG was determined in all M13HS hybrid clone cells (Fig. 2c-g). In the majority of the M13HS hybrids, SOX9 was localized in the nucleus and SLUG in the cytoplasm (Fig. 2c-g, green arrowheads), whereby in a few hybrid cells, a nuclear co-localization of both stemness factors was also observed (Fig. 2c-g; white arrows).

### Each M13HS hybrid clone exhibits a discrete population of ALDH1-positive cells

**Fig. 3:**
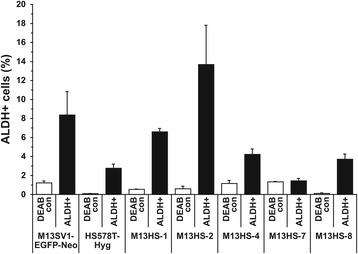
M13HS hybrid clone cells harbor a unique population of ALDH-positive cells. The frequency of ALDH-positive cells within the investigated cell lines was determined by flow cytometry using AldeRed fluorescent dye. Shown is the mean relative frequency of ALDH-positive cells of three independent experiments analyzing cells of different passages. Control cells were treated with the ALDH inhibitor DEAB

The AldeRed assay was performed to determine the frequency of ALDH1-positive cells within the analyzed cell lines, since ALDH1 is a well-known marker of normal and malignant human mammary stem cells [28, 29]. The population of ALDH1-positive cells within M13SV1-EGFP-Neo breast epithelial cells was approximately 8.4 ± 2.5%, whereas ALDH1 expression was determined in approximately 2.8 ± 0.4% of HS578T-Hyg human breast cancer cells (Fig. 3). M13HS hybrid clone cells varied markedly in the frequency of ALDH1-positive cells. For instance, the highest ALDH1 expression was determined in the M13HS-2 hybrid clone cells (13.7 ± 4.1%; Fig. 3), whereas virtually no ALDH1-positive cells were found in the M13HS-7 hybrid cells (DEAB control cells: 1.3 ± 0.1% vs. ALDH1-positive cells: 1.4 ± 0.3%; Fig. 3). The frequency of ALDH1-positive cells in the M13HS-1, M14HS-4 and M13HS-8 hybrid cell clones varied between 3.7 ± 0.6% (M13HS-8) and 6.6 ± 0.4% (M13HS-1; Fig. 3) indicating that each M13HS hybrid clone exhibits a unique population of ALDH1-positive cells.

### M13HS hybrid cell clones possess an increased colony forming capacity

**Fig. 4:**
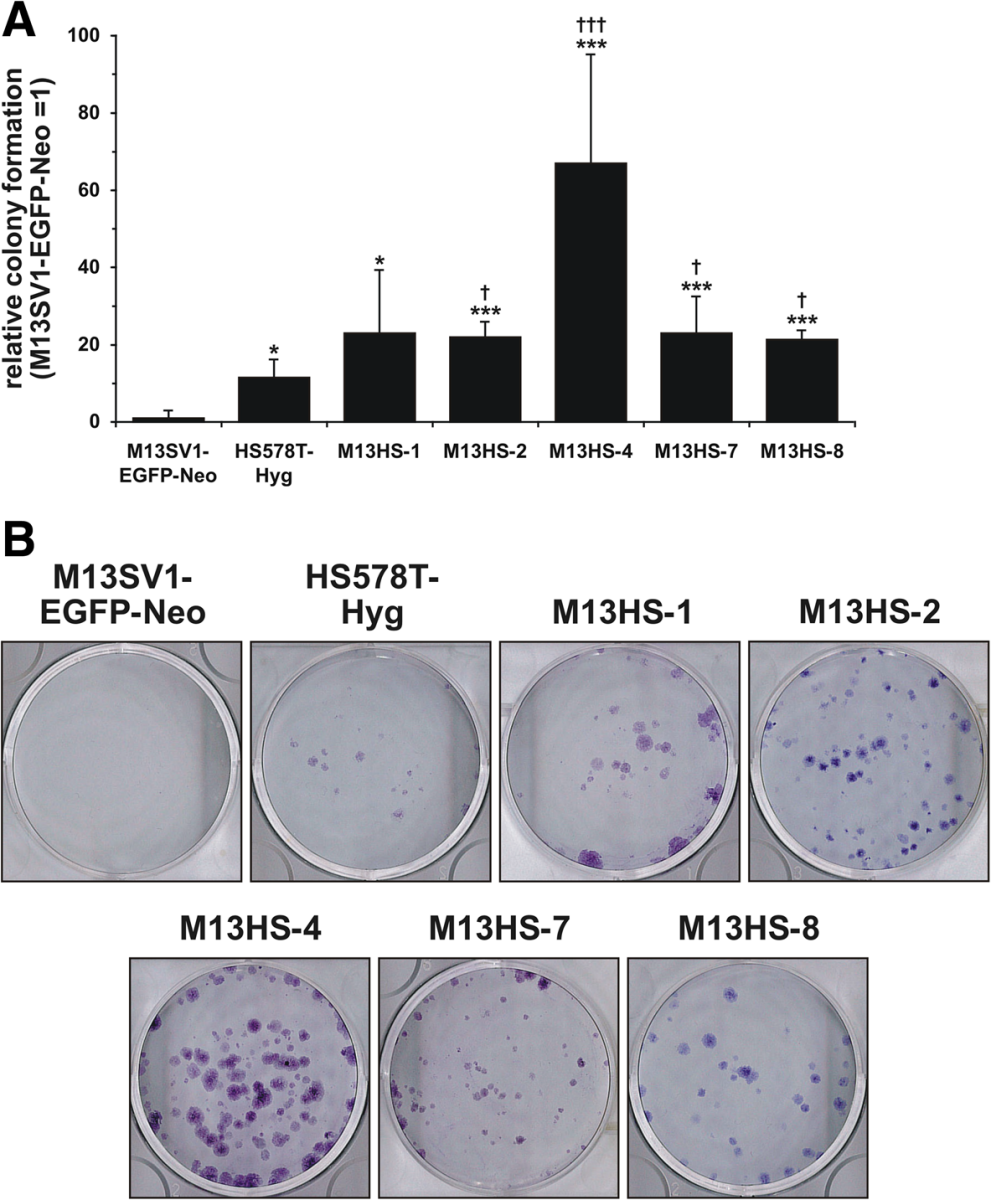
M13HS hybrid clone cells exhibit an increased colony formation capacity. Cells (2×10^2^ per well) were seeded in 6-well plates and were cultivated in complete media for 10–14 days. Growing colonies were stained with crystal violet solution. **a** Shown is the mean relative colony formation capacity of three independent experiments in relation to that of the M13SV1-EGFP-Neo human breast epithelial cells, which was set to 1. Statistical analysis was performed using an unpaired two-tailed Student’s t-test: * = *p* < 0.05, ** = *p* < 0.01, *** = *p* < 0.001 (vs. M13SV1-EGFP-Neo cells); † = *p* < 0.05, †† = *p* < 0.01, ††† = *p* < 0.001 (vs. HS578T-Hyg cells). **b** Shown are representative images of colonies derived from the indicated cell lines

Next, the colony formation capability of the parental cells and M13HS hybrid clone cells was analyzed. Data are summarized in Fig. 4 and clearly show that all hybrid cell clones exhibited a significantly increased colony forming capacity in comparison to the parental M13SV1-EGFP-Neo human breast epithelial cells and the HS578T-Hyg human breast cancer cells (Fig. 4a). The colony forming capacity of M13HS hybrid cell clones 1, 2, 7, and 8 was rather similar among each other and was significantly increased compared to the M13SV1-EGFP-Neo breast epithelial cells and the HS578T-Hyg breast cancer cells (Fig. 4a). A markedly increased colony formation capacity was determined for the M13HS-4 hybrid cells, which was approximately 67-fold higher than the M13SV1-EGFP-Neo breast epithelial cells and approximately 6-fold higher than the HS578T-Hyg breast cancer cells (Fig. 4a).

### M13HS hybrid cell clones possess an increased mammosphere forming capacity

**Fig. 5:**
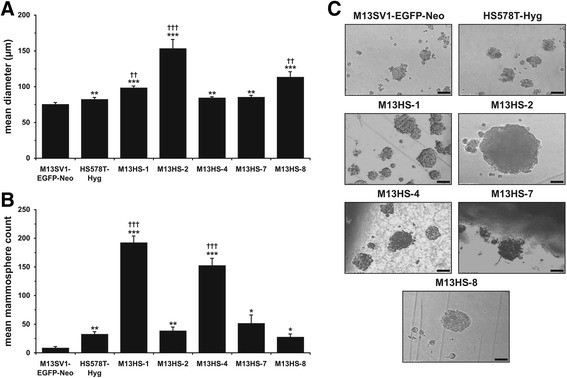
M13HS hybrid clone cells possess a unique mammosphere formation capacity. **a** Mean diameter of originated mammospheres, **b** mean mammosphere count. Shown are the means of at least five independent experiments using cells of different passages. Statistical analysis was performed using an unpaired two-tailed Student’s *t-test*: * = *p* < 0.05, ** = *p* < 0.01, *** = *p* < 0.001 (vs. M13SV1-EGFP-Neo cells); † = *p* < 0.05, †† = *p* < 0.01, ††† = *p* < 0.001 (vs. HS578T-Hyg cells). **c** Shown are representative images of mammospheres derived from the indicated cell lines. Note the markedly increased size of M13HS-2 mammospheres. Bar = 100 μm

In addition to the colony formation assay, the parental cells’ and M13HS hybrid clone cells’ ability to induce mammospheres was investigated. The M13SV1-EGFP-Neo human breast epithelial cells only exhibited a rather weak mammosphere formation capacity. A mean of 3 ± 2 M13SV1-EGFP-Neo mammospheres were formed with an average diameter of 70 ± 7 μm (Fig. 5a, b). The average diameter of the HS578T-Hyg mammospheres was approximately 82 ± 3 μm, and thus comparable to that of the M13SV1-EGFP-Neo human breast epithelial cells (Fig. 5a). However, significantly more mammospheres originated from the HS578T-Hyg human breast cancer cells (39 ± 25; *p* < 0.01; Fig. 5b). Each hybrid cell clone exhibited a unique mammosphere formation capacity. Interestingly, the capacity of the M13HS hybrid cell clones for forming mammospheres was not correlated to mammosphere size. For instance, the largest mammospheres were derived from the M13HS-2 and M13HS-8 hybrid clones, with a maximum diameter of single mammospheres of up to 460 μm (M13HS-2) and 247 μm (M13HS-8; Fig. 5a), whereas the mean mammosphere counts of the M13HS-2 and M13HS-8 hybrid clones were approximately 38 ± 7 and 27 ± 6, respectively, and thus comparable to the HS578T-Hyg breast cancer cells (Fig. 5b). In contrast, the mean diameter of the M13HS-1 and M13HS-4 hybrid clones was approximately 98 ± 3 μm and 84 ± 2 μm, respectively, whereas on average, there were 192 ± 12 and 152 ± 13 mammospheres derived from the M13HS-1 and M13HS-4 hybrid clone cells (Fig. 5a, b).

### Mammospheres derived from M13HS hybrid clone cells revealed SLUG and SOX9 expression and cellular distribution patterns similar to single cells

**Fig. 6:**
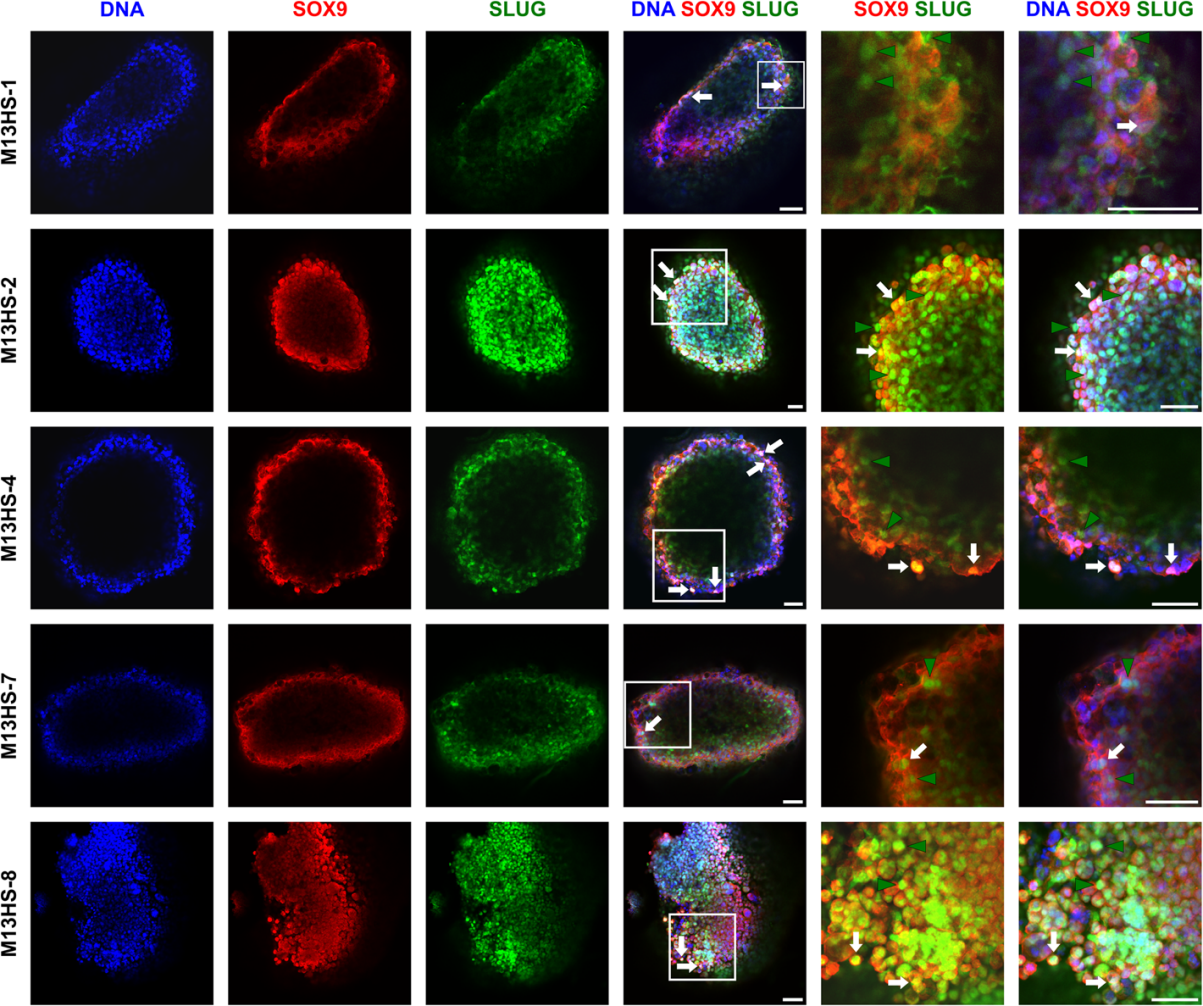
Confocal laser scanning microscopy of intracellular SOX9 and SLUG localization within mammospheres. Mammospheres were grown for 10 days, fixed, permeabilized and stained for SOX9, SLUG and DNA. In accordance with Fig. 2, *white arrows* indicate cells with a nuclear co-localization of SOX9 and SLUG. *Green arrowheads* indicate cells with nuclear SOX9 and cytosolic SLUG. Due to the size of the mammospheres, the inner regions remain unstained. Nonetheless, cells with a nuclear co-localization of SOX9 and SLUG could be found in the periphery of the mammospheres. The *white square* indicates areas that were further analyzed at a higher magnification. Bar = 50 μm

Next, the expression and intracellular localization of SLUG and SOX9 in mammospheres derived from M13SH hybrid clone cells were analyzed. Because whole mammospheres were analyzed with a diameter of up to 400 μm, the inner parts remained unstained and thus only the cells in the periphery could be investigated. Co-expression of SLUG and SOX9 was observed in all cells of M13HS hybrid clone-derived mammospheres (Fig. 6). In the majority of the hybrid clone cells, SOX9 was expressed in the nucleus and SLUG was localized in the cytosol (Fig. 6, green arrowheads). Nonetheless, some M13HS hybrid clone cells showed a nuclear co-localization of SOX9 and SLUG (Fig. 6, white arrows).

### M13HS hybrid cell clones exhibit an epithelial-mesenchymal transition (EMT) phenotype

**Fig. 7:**
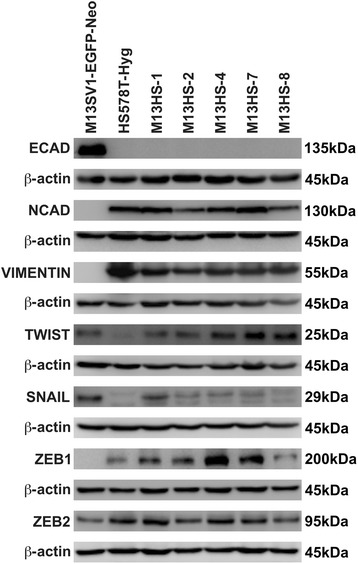
Expression of EMT-related marker proteins in M13HS hybrids and parental cells. Western blot data reveal an epithelial phenotype of M13SV1-EGFP-Neo breast epithelial cells, indicated by E-CADHERIN (ECAD) expression, but lack of N-CADHERIN (NCAD), VIMENTIN and ZEB1. In contrast, the HS578T-Hyg breast cancer cells and all M13HS hybrids exhibit a mesenchymal phenotype due to expression of NCAD, VIMENTIN and ZEB1, and a lack of ECAD. Interestingly, SNAIL and TWIST, which have both been associated with EMT, show higher expression in the M13SV1-EGFP-Neo breast epithelial cells than in the HS578T-Hyg breast cancer cells. Shown are the means of at least three independent experiments

Because SLUG belongs to the group of transcription factors involved in the EMT process, we also analyzed the expression of classical EMT markers, such as E-CADHERIN, N-CADHERIN and VIMENTIN, as well as other EMT-transcription factors including TWIST, SNAIL, ZEB1 and ZEB2 (Fig. 7).

In brief, the M13SV1-EGFP-Neo human breast epithelial cells exhibited an epithelial phenotype, whereas the HS578T-Hyg human breast cancer cells and all M13HS hybrid cell clones possessed a mesenchymal phenotype. The M13SV1-EGFP-Neo cells were positive for E-CADHERIN, but negative for N-CADHERIN, VIMENTIN and ZEB1 expression (Fig. 7). In contrast, the HS578T-Hyg cells and M13HS hybrid clones were positive for the mesenchymal markers N-CADHERIN, VIMENTIN and ZEB1, but negative for the epithelial marker E-CADHERIN (Fig. 7). Interestingly, the M13SV1-EGFP-Neo cells expressed higher levels of SNAIL and TWIST than HS578T-Hyg breast cancer cells, which have both been associated with EMT (Fig. 7) [30]. In contrast, lower levels of ZEB2 (another EMT marker [30]) were detected in the M13SV1-EGFP-Neo cells compared to the HS578T-Hyg breast cancer cells (Fig. 7).

All M13HS hybrid clone cells revealed a unique expression pattern of the EMT transcription factors TWIST, SNAIL, ZEB1 and ZEB2 (Fig. 7). For instance, TWIST was markedly expressed in the M13HS-7 and M13HS-8 hybrid clone cells, but rather moderate in the M13HS-1 and M13HS-2 hybrid clone cells (Fig. 7). In contrast, the highest ZEB1 expression levels were observed in the M13HS-4 and M13HS-7 hybrid clone cells, whereas only a faint ZEB1 expression was determined in the M13HS-8 hybrid clone cells (Fig. 7). Expression of SNAIL was determined in all M13HS hybrid cell clones, where the highest SNAIL expression was found in the M13HS-1 hybrid clone cells and the lowest SNAIL expression in the M13HS-8 hybrid clone cells (Fig. 7).

### Cell migration studies

**Fig. 8:**
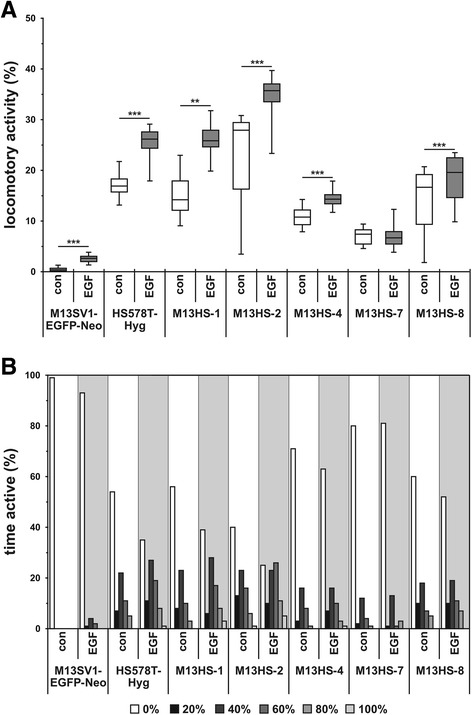
Cell migration data. Cell migration was analyzed using the three-dimensional collagen matrix migration assay in combination with computer-assisted cell-tracking. **a** The locomotor activity of the cell population is displayed as a box plot. **b** The time active of all single cells analyzed in this study is shown as a bar chart. Non-migrating cells possess a time active of 0%. With the exception of the M13HS-7 hybrid clone cells, each cell line responded to EGF with an increased locomotor activity. Thereby, the time active data reveal that the EGF-induced cell migration was attributed to an increased number of moving cells and a prolonged time of active movement. Shown are the means of at least three independent experiments. Statistical analysis was performed using a two-tailed Mann-Whitney U test. ** = *p* < 0.01; *** = *p* < 0.001

Since all hybrid clone cells exhibit a mesenchymal phenotype, we additionally investigated the cells’ migratory activity in response to EGF. With the exception of M13HS-7 hybrid clone cells (Fig. 8a, b), all hybrid clone cells responded to EGF stimulation with an increased migratory activity, which is consistent with previously published data [22]. The M13SV1-EGFP-Neo breast epithelial cells revealed a rather weak spontaneous migratory activity with a median of 0.42% (Fig. 8a). Nonetheless, the locomotor activity was increased to 2.64% (median) in response to EGF stimulation (Fig. 8a). The HS578T-Hyg breast cancer cells and M13HS-1 hybrid cells exhibited a similar migratory phenotype, and the EGF-induced migration was attributed to both an enhanced number of moving cells as well as an increased time of active movement (Fig. 8a, b). Of all the investigated cell lines, the M13HS-2 hybrid clone cells possessed the highest spontaneous locomotor activity (median 27.95%), which also applied to the cells’ migratory behavior in response to EGF (median 35.69%; Fig. 8a). The spontaneous migration of the M13HS-8 hybrid clone cells was comparable to the HS578T-Hyg breast cancer cells and the M13HS-1 hybrid cells. However, compared to the HS578T-Hyg and M13HS-1 cells, the migratory activity of the M13HS-8 hybrid clone cells was only moderately increased in response to EGF stimulation (Fig. 8a). Both the M13HS-4 and M13HS-7 hybrid clone cells showed a weak spontaneous locomotor activity that was between the spontaneous migratory activities of the parental cells (Fig. 8a). The median EGF-induced migratory activity of the M13HS-4 hybrid clone cells was slightly higher than untreated cells (control: 10.79% vs. 100 ng/mL EGF: 14.29%; Fig. 8a). An increased migratory activity of the cells in response to EGF was attributed to both an increased number of moving cells and an increased time of active movement, indicating that EGF induced cell migration in non-moving cells and caused moving cells to migrate for a longer time (Fig. 8b).

## Discussion

In the present study, we demonstrated that M13HS hybrid clone cells, which originated from spontaneous cell fusion events between human M13SV1-EGFP-Neo breast epithelial cells exhibiting stem cell properties and human HS578T-Hyg breast cancer cells [15, 22], putatively exhibit CS/IC properties and an EMT phenotype.

M13SV1 human breast epithelial cells were generated from primary type I human breast epithelial cells (HBECs) by SV40 immortalization and were considered to exhibit stem cell properties [31–33]. Kao et al. demonstrated that M13SV1 cells exhibited the following characteristics: deficient in gap junctional intercellular communication (GJIC), which has been suggested as a characteristic of putative stem cells; expressed luminal markers such as epithelial membrane antigen (EMA), CK8 (our data and [34]) and CK18, but not CK14; and able to give rise to type II human breast epithelial cells, which revealed a rather basal phenotype due to expression of CK14 and α_6_-integrin [31]. Western blot analysis revealed a low CK14 expression in the M13SV1-EGFP-Neo human breast epithelial cells suggesting that M13SV1 cells may comprise a small population of M13SV1-derived type II human breast epithelial cells. In contrast, the HS578T-Hyg human breast cancer cells lacked CK8 expression, but revealed a high expression of CK14, which is in accordance with the cells’ basal-like B phenotype [35]. All five M13HS hybrid clones revealed a dual expression of CK8 and CK14. Immunohistochemistry data showed that the M13HS-2 and M13HS-8 hybrid clone cells truly co-expressed both cytokeratins (data not shown), indicating that both hybrid clone cells do not consist of two distinct subpopulations either expressing CK8 or CK14. A subpopulation of CK5/CK14 basal breast carcinomas also showed luminal CK8/CK18 positivity; such carcinomas have been termed basoluminal tumors [36]. Moreover, lower levels of VIMENTIN were detected in basoluminal tumors in comparison to basal tumors [36]. This finding is in accordance with the Western blot data showing clearly showing lower VIMENTIN expression levels in the M13HS hybrid clone cells than in the HS578T-Hyg breast cancer cells. However, other markers that have been associated with basoluminal tumors, such as Ki67 or EGFR gene amplification [36], have not yet been analyzed in the M13HS hybrid clone cells, and thus it remains unclear whether these hybrid cells truly exhibit a basoluminal phenotype.

In accordance with the dual CK5 and CK14 expression, all hybrid clone cells also revealed a co-expression of SOX9 and SLUG, which cooperatively determine the stem cell state of normal human mammary cells and human breast cancer cells [26]. Nuclear co-localization of both transcription factors was observed in the parental M13SV1-EGFP-Neo human breast epithelial cells and all M13HS hybrid clone cells (both single cells and cells within mammospheres). However, in the majority of the cells, SLUG was present in the cytoplasm and SOX9 was present in the nucleus. Phosphorylation of SLUG at position Ser100/104 by glycogen synthase kinase-3β (GSK-3β) has been associated with translocation to the cytosol and presumably proteasomal degradation, whereas further phosphorylation at Ser92/96 has been shown to prevent SLUG from degradation concomitant with its accumulation in the cytosol [37]. Whether a similar mechanism might be responsible for cytosolic SLUG localization in the analyzed cells remains to be elucidated. In any case, the co-expression and nuclear co-localization of SLUG and SOX9 indicates that the M13HS hybrid clone cells may exhibit stem cells properties. However, the M13HS hybrid clone cells varied markedly among each other concerning ALDH1 expression, colony formation capacity and mammosphere formation capacity. Thus, the capability for colony formation and mammosphere formation may not only be attributed to the expression of transcription factors and ALDH1, but most likely also to other mechanisms. For instance, Liu and colleagues demonstrated that the self-renewal of normal and malignant human mammary stem cells is regulated by Hedgehog signaling and Bmi-1 [38]. Activation of Hedgehog signaling was further correlated with an increased mammosphere-initiating cell number and mammosphere size, whereas inhibition of these pathways resulted in a reduction of these effects [38]. Increased mRNA levels of Bmi-1 as well as OCT4, NANOG, NOTCH1, ALDH1, SOX2, and CD133 were also found in spontaneously formed hybrid cells derived from non-small cell lung cancer cells and bone-marrow-derived mesenchymal stem cells, which also possessed an increased pneumosphere-forming capacity and tumor-forming ability [20]. Elevated mRNA levels of OCT4, NANOG, SOX2, LIN28, and CD133 have also been identified in tumorigenic hybrid cells derived from mesenchymal stem cells and gastric cancer cells [19]. Expression of OCT4 or transmembrane delivery of OCT4 protein promotes dedifferentiation of melanoma cells to CS/IC-like cells possessing an increased tumorsphere-formation capacity, an enhanced tumorigenic capacity and increased expression levels of endogenous OCT4, NANOG and KLF4 [39]. OCT4-induced CS/IC features in melanoma cells were reverted by RNAi-mediated knock-down of OCT4 [39]. Similar findings were reported for the knock-down of Oct4 and Nanog in human MDA-MB-231-derived breast CS/ICs that were correlated with a reduced tumorigenicity and drug resistance [40], indicating a putative role of OCT4 and NANOG in CS/IC biology. M13SV1 human breast epithelial cells do express OCT4 [33], suggesting that M13HS hybrid clone cells might also be positive for this stemness transcription factor.

Promotion of CS/IC self-renewal and mammosphere growth of human breast tumors, including triple negative breast cancers (TNBC), has been further associated with IL-6, IL-8 and TGF-β [41–45]. Charafe-Jauffret et al. demonstrated that IL-8 increased the mammosphere formation and the ALDEFLUOR-positive population in human breast cancer cell lines in a dose-dependent manner [41]. Furthermore, ALDEFLUOR-positive breast cancer cells exhibited an increased invasion capacity that was further positively triggered by IL-8, suggesting that IL-8 might also play a role in cancer metastases [41]. These data are supported by results indicating that concurrent inhibition of IL-6 and IL-8 expression in TNBC cells dramatically inhibited colony formation and cell survival in vitro and stanched tumor engraftment and growth in vivo [44]. Moreover, IL-6 and IL-8 expression levels were correlated to patient survival time, suggesting a rationale for dual inhibition of IL-6/IL-8 signaling as a therapeutic strategy to improve outcomes for patients with TNBCs [44]. TGF-β facilitates breast cancer stem cell self-renewal and expansion in TNBCs via induction of cyclooxygenase-2 (COX-2) expression [45]. Knock-down of COX-2 expression or inhibition of COX-2 activity using a pharmacological inhibitor strikingly blocked TGF-β-induced tumorsphere formation, enrichment of CD24^low^CD44^high^, ALDH^+^ breast cancer stem cells and breast cancer stem cell self-renewal [45]. HS578T human breast cancer cells have been classified as triple negative [46], suggesting that M13HS hybrid clone cells might also exhibit this phenotype. In fact, M13HS hybrid clones express low levels of HER2 [22] and lack estrogen receptor expression (unpublished data). In this regard, it would be of interest to investigate whether the differential mammosphere formation capacity, as well as mammosphere size, might be related to an altered Hedgehog signaling and/or differentially regulated autocrine IL-6 and IL-8 loops in M13HS hybrid clone cells.

The impact of inflammatory factors, such as IL-6, might also be of interest in the context of breast cancer cells possessing a hybrid epithelial/mesenchymal (E/M) or a partial EMT phenotype [47]. Both EMT and its reverse, mesenchymal-to-epithelial transition (MET), are hallmarks of cancer metastasis [47, 48]. However, while transitioning between the epithelial and mesenchymal phenotypes, cells can also attain a hybrid E/M phenotype and thus have mixed epithelial (e.g., adhesion) and mesenchymal (e.g., migration) properties that could enable cancer cells to move collectively as clusters through the connective tissue and even through the circulation [47]. Moreover, the hybrid E/M phenotype has been further associated with stemness in all breast cancer subtypes, and expression of a mixed E/M gene signature is correlated to the poorest outcomes in luminal and basal breast cancer patients [49]. Inflammation might stabilize a mixed E/M hybrid phenotype and even stemness due to induction of a self-perpetuating Notch-Jagged and NF-κB signaling loop [47, 50], which in turn coincides with data showing that IL-6 (and IL-8) promotes CS/IC self-renewal and mammosphere growth [41–45]. Grosse-Wilde et al. demonstrated that hybrid E/M cells displayed an increased mammosphere formation capacity and produced more ALDH1-positive progenies than E or M cells alone [49]. However, a marked increase in the mammosphere formation capacity was only observed for the M13HS-1 and M13HS-4 hybrid clone cells, whereby the frequency of ALDH1-positive M13HS-1 and M13HS-4 hybrid cells was rather moderate. In contrast, the M13HS-2 hybrid cells exhibited the highest frequency of ALDH1-positive cells of all the M13HS hybrid clones but possessed a rather weak mammosphere formation capacity. Thus, the mammosphere formation capacity and the ALDH1 frequency of M13HS hybrid clone cells are most likely not related to each other, suggesting that M13HS hybrid clone cells may not exhibit a mixed E/M phenotype. This would further correlate with preliminary flow cytometry data showing that M13HS hybrid clone cells possess the breast cancer stem cell phenotype CD24^−/low^CD44^+^ [51], which is conflicting to data of Grosse-Wilde et al. demonstrating that hybrid E/M cells that possess putative CS/IC properties were CD24^+^/CD44^+^ [49]. Likewise, Western blot data clearly indicate that M13HS hybrid clone cells do not express E-CADHERIN, but N-CADHERIN and VIMENTIN instead, suggesting that the cells likely exhibit a mesenchymal than a mixed E/M phenotype. However, no single-cell analysis was performed in this study and only a few EMT marker proteins were analyzed. Hence, it cannot be ruled out that single hybrid clone cells exhibiting a mixed E/M phenotype do exist.

The Western blot data revealed that all M13HS hybrid clone cells co-expressed SNAIL, ZEB1 and ZEB2. ZEB and SNAIL belong to a family of well-known EMT transcription factors [52], whereby ZEB expression is induced by SNAIL [53]. Both SNAIL and ZEB might induce and maintain a mixed E/M phenotype if both transcription factors were co-expressed in a certain ratio [47]. The observation that the mixed E/M phenotype has been associated with stemness in all breast cancer subtypes [49] suggests a putative role for SNAIL (and ZEB) in the regulation of the stem cell state of breast CS/ICs. This assumption is in accordance with recent findings indicating a more pivotal role of SNAIL in breast cancer than previously thought [27]. SNAIL knock-down in human MDA-MB-231 breast cancer cells was associated with the induction of MET, loss of ZEB1 and reactivation of E-CADHERIN [27], indicating that cells have adopted an E phenotype. Moreover, knock-down of SNAIL strongly impaired the capacity of MDA-MB-231 breast cancer cells to induce primary tumors and metastases in a xenograft model [27]. Given that primary tumors (and metastases) arise from CS/ICs [54], these findings likely indicate that a lack of SNAIL might be associated with a loss of stemness in breast CS/ICs, possibly due to conversion from a mixed E/M phenotype to an E phenotype. Thus, in ongoing studies, the role of SNAIL in M13HS hybrid clone cells should be clarified, which should also include gene expression studies to elucidate whether single M13HS hybrid clone cells do exhibit a putative mixed E/M phenotype.

## Conclusions

The data presented here indicate that the fusion of human breast cancer cells and human breast epithelial cells exhibiting stem cell properties could give rise to hybrid clone cells possessing characteristics of CS/ICs, such as an increased frequency of ALDH1-positive cells and an increased capability of forming colonies and mammospheres. In summary, these data are in accordance with previously published data revealing that CS/ICs or CS/IC-like cells could originate from cell fusion events between cancer cells and stem cells or stem-like cells [11, 18–20] or even from normal, non-transformed epithelial cells [21]. Moreover, these findings indicate that the heterogeneity of the CS/IC pool is most likely attributable not only to clonal evolution [54] but also to cell fusion. In this regard, we have already postulated that so-called second-line or third-line CS/ICs may originate at later stages in the life of a tumor from fusion events between tumor cells and bone marrow-derived cells or tissue stem cells [55]. Were this assumption to be true, cell fusion might not only increase the heterogeneity of the tumor tissue but also foster the heterogeneity of the CS/IC pool inside a tumor, which might be associated with a considerably accelerated tumor progression.

## Additional files

Additional file 1: Figure S1. M13HS hybrid clone cells possess an increased mean chromosomal number. Shown are chromosome numbers of at least 15 metaphase spreads. Note that each hybrid clone exhibit a unique mean chromosomal number. (TIFF 1248 kb)

Additional file 2: Figure S2. M13HS hybrid clones are mononuclear. The morphology of M13HS hybrid clone cells resembles more to the morphology of parental HS578T-Hyg human breast cancer cells than to M13SV1-EGFP-Neo breast epithelial cells. Please note that the EGFP fluorescence of M13SV1-EGFP-Neo cells and M3HS hybrid clone cells was not visualized here due to the much brighter fluorescence of SYTOX Green. Shown are representative images. Bar = 20 μm. (TIFF 12426 kb)

## Abbreviations

ALDH1: Aldehyde dehydrogenase 1
AML: Acute myeloid leukemia
BSA: Bovine serum albumin
CK: Cytokeratin
COX-2: cyclooxygenase-2
CS/ICs: Cancer stem/initiating cells
DEAB: Diethylamino benzaldehyde
E/M: Epithelial/mesenchymal
EGF: Epidermal growth factor
EMT: Epithelial-mesenchymal-transition
EMT: Mesenchymal-epithelial-transition
FCS: Fetal calf serum
FGF: Fibroblast growth factor
HST: Heterokaryon-to-synkaryon transition
NOD/SCID: Non-obese diabetic mice with severe combined immunodeficiency disease
PBS: Phosphate buffered saline
PFA: Paraformaldehyde
poly-HEMA: Poly-(2-hydroxyethyl-methacrylate)
PVDF: Polyvinyl difluoride
rCSCs: Recurrence cancer stem cells
RT: Room temperature
SDS-PAGE: Sodium dodecyl sulfate-polyacrylamide gel electrophoresis
SP cells: Side population cells
STR analysis: Short-tandem-repeat analysis
TBS-T: Tris-buffered saline with 1% Tween 20
TNBC: Triple negative breast cancer
