# Supplementary figures and images for: Hybrid clone cells derived from human breast epithelial cells and human breast cancer cells exhibit properties of cancer stem/initiating cells
Source: BMC Cancer. 2017 Aug 2;17:515. doi: 10.1186/s12885-017-3509-9 (PMC5541689; doi:10.1186/s12885-017-3509-9)

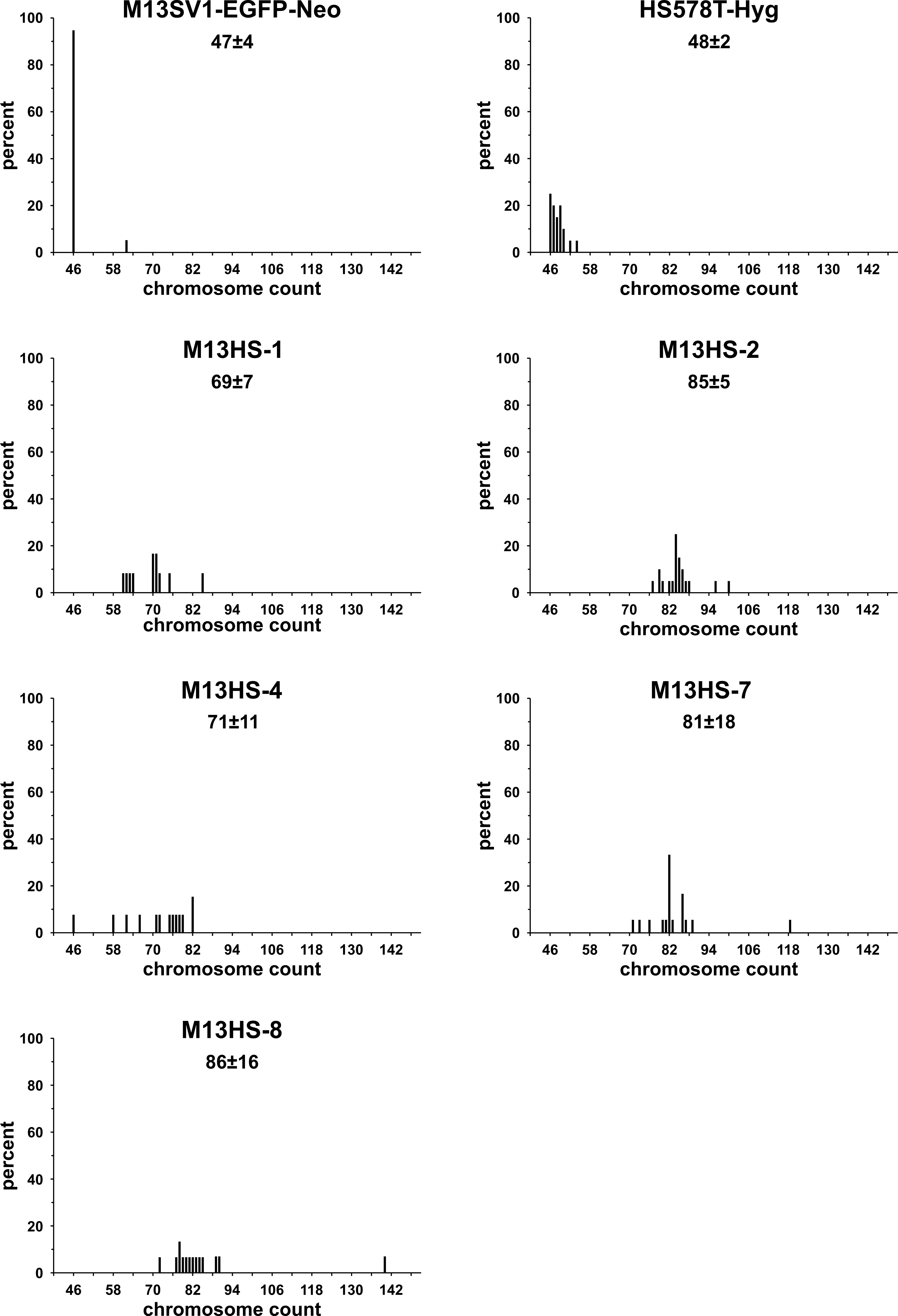

Supplement: Supplementary file 1 — M13HS hybrid clone cells possess an increased mean chromosomal number. Shown are chromosome numbers of at least 15 metaphase spreads. Note that each hybrid clone exhibit a unique mean chromosomal number. (TIFF 1248 kb) [file 12885_2017_3509_MOESM1_ESM.tif]

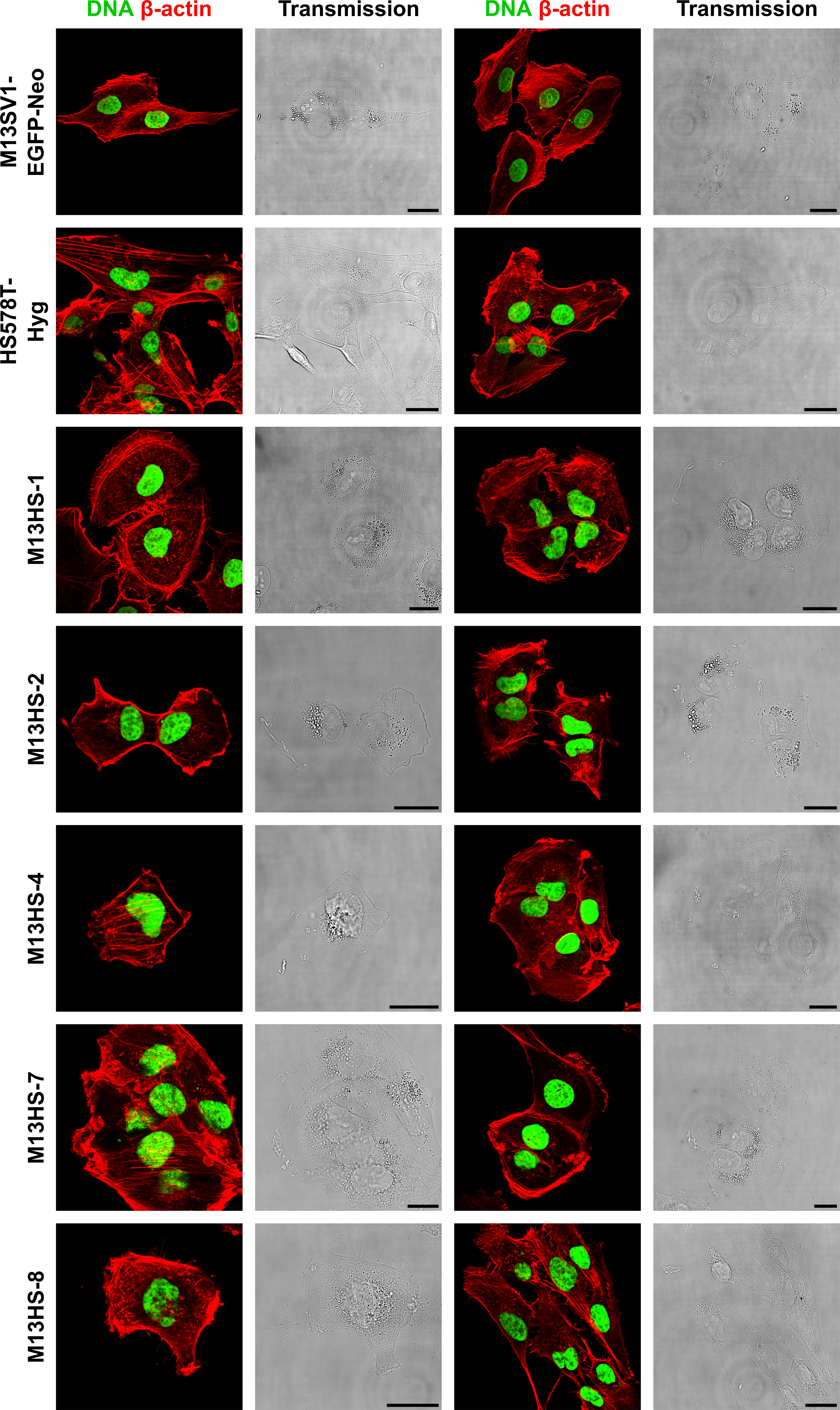

Supplement: Supplementary file 2 — M13HS hybrid clones are mononuclear. The morphology of M13HS hybrid clone cells resembles more to the morphology of parental HS578T-Hyg human breast cancer cells than to M13SV1-EGFP-Neo breast epithelial cells. Please note that the EGFP fluorescence of M13SV1-EGFP-Neo cells and M3HS hybrid clone cells was not visualized here due to the much brighter fluorescence of SYTOX Green. Shown are representative images. Bar = 20 μm. (TIFF 12426 kb) [file 12885_2017_3509_MOESM2_ESM.tif]
